# Immunomodulation Targeting Abnormal Protein Conformation Reduces Pathology in a Mouse Model of Alzheimer's Disease

**DOI:** 10.1371/journal.pone.0013391

**Published:** 2010-10-13

**Authors:** Fernando Goñi, Frances Prelli, Yong Ji, Henrieta Scholtzova, Jing Yang, Yanjie Sun, Feng-Xia Liang, Regina Kascsak, Richard Kascsak, Pankaj Mehta, Thomas Wisniewski

**Affiliations:** 1 Department of Neurology, New York University School of Medicine, New York, New York, United States of America; 2 Department of Pathology, New York University School of Medicine, New York, New York, United States of America; 3 Department of Psychiatry, New York University School of Medicine, New York, New York, United States of America; 4 Image Core Facility, New York University School of Medicine, New York, New York, United States of America; 5 New York State Institute for Basic Research in Developmental Disabilities, Staten Island, New York, United States of America; 6 Department of Immunology, School of Chemistry, University of Uruguay, Montevideo, Uruguay; Massachusetts General Hospital and Harvard Medical School, United States of America

## Abstract

Many neurodegenerative diseases are characterized by the conformational change of normal self-proteins into amyloidogenic, pathological conformers, which share structural properties such as high β-sheet content and resistance to degradation. The most common is Alzheimer's disease (AD) where the normal soluble amyloid β (sAβ) peptide is converted into highly toxic oligomeric Aβ and fibrillar Aβ that deposits as neuritic plaques and congophilic angiopathy. Currently, there is no highly effective treatment for AD, but immunotherapy is emerging as a potential disease modifying intervention. A major problem with most active and passive immunization approaches for AD is that both the normal sAβ and pathogenic forms are equally targeted with the potential of autoimmune inflammation. In order to avoid this pitfall, we have developed a novel immunomodulatory method that specifically targets the pathological conformations, by immunizing with polymerized British amyloidosis (pABri) related peptide which has no sequence homology to Aβ or other human proteins. We show that the pABri peptide through conformational mimicry induces a humoral immune response not only to the toxic Aβ in APP/PS1 AD transgenic mice but also to paired helical filaments as shown on AD human tissue samples. Treated APP/PS1 mice had a cognitive benefit compared to controls (p<0.0001), associated with a reduction in the amyloid burden (p = 0.0001) and Aβ40/42 levels, as well as reduced Aβ oligomer levels. This type of immunomodulation has the potential to be a universal β-sheet disrupter, which could be useful for the prevention or treatment of a wide range of neurodegenerative diseases.

## Introduction

The diagnostic neuropathological lesions of AD are the accumulation of amyloid β (Aβ) as neuritic plaques and congophilic angiopathy, as well as aggregation of abnormally phosphorylated tau in the form of neurofibrillary tangles (NFTs) [1]. AD is the most common of the neurodegenerative protein conformational disorders, which include diffuse Lewy body disease (DLBD), Parkinson's disease (PD), prion diseases, and frontotemporal lobar degeneration (FTLD). In each of these disorders a normal self-protein/peptide, which has a physiological function, undergoes a conformational change to a pathological conformer that has a high β-sheet content, is resistant to degradation and accumulates either in extracellular plaques or intracellular inclusion bodies, with the most toxic conformers being oligomeric [2]. In AD the normal soluble Aβ (sAβ) and tau are converted to Aβ and abnormally phosphorylated tau in NFTs, respectively. Eleven different proteins are known to accumulate as oligomers, plaques and/or intra-cellular inclusions in the CNS leading to various neurodegenerative diseases, with the most common being Aβ, phosphorylated tau, α-synuclein and TDP-43 [2]; [3]. Among patients with a clinical diagnosis of dementia, neuropathological examination reveals that in a majority of cases there is an accumulation of a mixture of different pathological protein conformers, with the most common mix being Aβ, phosphorylated tau and α-synuclein [4]. However, a continuum also exists between AD and FTLD associated pathology with some 23–34% of AD cases having TDP-43 inclusions [5]; [6]. One explanation for this frequent co-occurrence of age associated pathologies in a given patient's brain is that one type of pathological conformer can seed oligomerization/fibrillization in heterologous proteins which are prone to form amyloid, in what has been called abnormal conformational mimicry [7]; [8]. None of the conformational diseases has an effective therapy; however, immunotherapy has shown great promise for both AD and prion diseases, at least in mouse models [9]; [10]. However, potential toxic side effects with these immunological approaches targeting a self protein are autoimmune inflammatory complications. In the first human trial of active immunization for AD, 6% of patients developed encephalitis [11]. One possible way to avoid this is to use antibodies that specifically target the pathological conformer [12]. A few studies have tried conformation selective monoclonal antibodies therapeutically in AD mouse models and found this to have beneficial effects [13]–[15]. However, a major disadvantage to passive immunization for chronic neurodegenerative disorders would be the need for multiple infusions and the risk of developing anti-idiotypic immunity, which would limit efficacy and be associated with toxicity.

In the present study we sought to develop therapeutic immunomodulation through a conformation selective active immunization approach and test it therapeutically in an AD mouse model. This is an approach, which to our knowledge has not been tried previously. In this novel active immunomodulation approach, we used a polymerized British amyloidosis (ABri) related peptide in a predominantly β-sheet, oligomeric form. ABri is a rare form of familial human amyloidosis associated with a missense mutation in a stop codon resulting in the transcription of an intronic sequence, leading to production of a highly amyloidogenic protein with a carboxyl terminus that has no sequence homology to any other native human protein, including Aβ [16]; [17]. We hypothesized that through conformational mimicry the polymerized ABri peptide could induce a conformation selective immune response that will recognize Aβ (as well as other potentially amyloidogenic proteins such as phosphorylated tau). Such an immunostimulatory approach would have a reduced risk of inducing auto-immune complications as it is specific to a pathological conformer and the immunogen has no sequence homology to any known mammalian protein/peptide. We tested this approach in an APP/PS1 AD mouse model [18] and evaluated for behavioral benefits and reductions in Aβ related pathology histologically and biochemically.

## Materials and Methods

### A) Synthesis of Peptide

The 13 residue peptide corresponding to the carboxyl terminus of ABri (Cys-Ser-Arg-Thr-Val-Lys-Lys-Asn-Ile-Ile-Glu-Glu-Asn) was synthesized on an ABI 430A peptide synthesizer (AME Bioscience, Chicago, IL) at the Keck peptide synthesis facility at Yale University, CT, using a Vydac C18 preparative column, 2.5×30 cm (Vydac Separations, Hesperia, CA). Standard protocols for tBOC (tert-butyloxycarbonyl) chemistry were used. The peptide was subsequently cleaved from the resins using hydrofluoric acid and purified by high-pressure liquid chromatography (HPLC) on a Vydac C18 preparative column using linear gradients from 0–70% of acetonitrile in 0.1% trifluoroacetic acid. Mass spectroscopy of the lyophilized end-product was used to verify the expected molecular weight.

### B) Polymerization of the ABri Peptide and Assessment of Conformation

In order to make the 13 residue ABri peptide immunogenic and to potentially ensure a conformation specific immune response, the peptide was first subjected to controlled polymerization using the following protocol: The peptide was dissolved at 3 mg/ml, in 100 mM borate buffer saline (BBS), pH 7.4. Fresh 1% glutaraldehyde in BBS was prepared and added to the peptide to a final 5 mM glutaraldehyde concentration and incubated in an Eppendorf block shaker at 800 rpm and 56°C for 16 hrs. The solution was then quenched with 0.5 M glycine to make the solution 100 mM in glycine. After five minutes the solution was diluted 1∶3 with BBS, dialyzed against 2 mM BBS overnight at 4°C, aliquoted and lyophilized. To determine the degree of aggregation the original monomeric ABri peptide and polymerized ABri peptide (pABri) were electrophoresed on 12.5% SDS-polyacrylamide Tris-tricine gels under reducing conditions. Western blots were performed with a mouse anti-ABri polyclonal Ab [19] (1∶1,000 dilution) The secondary antibody (1∶2,000 dilution) was peroxidase-linked anti-rabbit IgG (Amersham Biosciences, Piscataway, NJ), and the immunoreactive material was visualized as chemoluminescence on X-ray film with an ECL detection kit (Pierce). For electron microscopic studies, the original and polymerized ABri peptides were incubated at 1 mg/ml in phosphate buffered saline, pH 7.4. 3 µl of sample was put onto a carbon coated 400 mesh Cu/Rh grid (Ted Pella Inc., Redding, CA) and stained with 1% uranyl acetate in distilled water (Polysciences, Inc, Warrington, PA). Stained grids were examined under a Philips CM-12 electron microscope (FEI; Eindhoven, The Netherlands) and photographed with a (1k x1k) digital camera (Gatan, Inc., Pleasanton, CA). For secondary structure analysis aliquots of the original ABri peptide and pABri were reconstituted in 5 mM Tris buffer (pH 7.0) to obtain a peptide concentration of 100 µM. Circular dichroism (CD) was measured on a Jasco J-720 spectropolarimeter (Easton, MD) equipped with a model CTC-344 circular temperature control system (Neslab Inc., Newington, NH) according to our previously described protocol [20]. The neural network algorithm (Softsec software; Softwood Inc., PA) was used to obtain percentages of different types of secondary structures of the analyzed peptides [21]; [22].

### C) Purification of paired helical filaments (PHF)

PHF were purified from the brain of a subject from the New York University Alzheimer's Disease Center brain bank, who fulfilled the National Institute on Aging-Reagan criteria for AD at autopsy by using a modification of a method previously reported [23],. Briefly, 30 gm of frontal cortex was homogenized in 75 ml of 50 mM Tris-buffered saline (TBS), pH 7.4 using an Ultra Turrox T25 tissue homogenize (IKA Works, Inc; Staufen, Germany). 75 ml of 20% sarcosyl in H_2_O was added to the sample and it was homogenized again. The homogenized material was centrifuged at 3,500 rpm in a Beckman GPR centrifuge and 6 ml aliquots of the supernatant were each layered over 1 ml TBS/0.1%SB3-14 and centrifuged in an Optima Max ultracentrifuge at 75,000 rpm for 2 hours at 20°C. Each pellet was resuspended by sonication in1 ml of 10%NaCl in TBS/0.1%SB3-14, followed by the addition of 6 ml of 10%NaCl in TBS/0.1%SB3-14 and centrifuged at 75,000 rpm for 1.5 hours at 20°C. The pellets were sonicated in 1 ml of 10%NaCl in TBS/0.1%SB3-14 followed by the addition of 6 ml of 10%NaCl in TBS/0.1%SB3-14, layered over 1 ml of 20% sucrose in 10%NaCl TBS/0.1%SB3-14 and centrifuged at 75,000 rpm for 1.5 hours at 20°C. The final pellets were resuspended in TBS/0.1%SB3-14 by sonication prior to use.

### D) Immunization of Mice

Animal studies were approved by the NYU School of Medicine Institutional Animal Care and Use Committee (protocol 070503-03) and were consistent with the recommendations of the American Veterinary Association. The pABri peptide was dissolved in sterile saline at 1 mg/ml and mixed 1∶1 with Aluminum Hydroxide (Alum) adjuvant (Brenntag Biosector, Denmark). Each mouse received a weekly subcutaneous injection of 100 µl of the preparation for 4 weeks followed by an inoculation a month later and two subsequent bimonthly injections. The last three inoculations used 25 µg of pABri per animal and the ratio of saline to alum ratio was changed to 9∶1. Two groups of 15 APP_ K670N/M671L_/PS1_ M146L_ (APP/PS1) Tg mice [18] were immunized with either pABri in saline with alum or saline with alum alone starting at the age of 2 months, with the mouse breeding and genotyping as previously described [24]. Animals were bled from the caudal vein one week after the 4th and 6th inoculations (T4 and T6, respectively) and at the time of sacrifice (TF). The blood was collected in heparinized tubes and plasma separated and stored at −80°C. Amyloid deposition in this mouse AD Tg model starts at about the age of 3 months [25]. At the age of 14 months the two groups of Tg mice were subject to behavioral testing using the radial arm maze with comparison to a third group of 15 mice which were age and sex matched, non-Tg littermate controls which had been given sc injections of saline/alum alone.

### E) Sensorimotor and Cognitive testing

Sensorimotor and cognitive testing were done as previously described [24]; [26]; [27]. Prior to testing, the mice were adapted to the room with lights on for 15 min. The main objective of performing these sensorimotor tasks was to verify that any treatment related effects observed in the cognitive tasks could not be explained by differences in sensorimotor abilities.

### Locomotor Activity

A Hamilton-Kinder Smart-frame Photobeam System was used to make a computerized recording of animal activity over a designated period of time. Exploratory locomotor activity is recorded in a circular open field activity chamber measuring (70×70 cm). A video camera mounted above the chamber automatically recorded horizontal movements in the open field in each dimension (i.e., x, y, and two z planes). Total distance was measured in centimeters (cm) traveled and is defined as sequential movement interruptions of the animal measured relative to the background. The duration of the behavior was timed for 15 min. Results were reported based on distance traveled (cm), mean resting time, and maximum velocity of the animal.

### Traverse Beam

This task tests balance and general motor coordination and function integration. Mice were assessed by measuring their ability to traverse a graded narrow wooden beam to reach a goal box specifically examining hind limb function. The mice were placed on a 1.1 cm wide beam 50.8 cm long suspended 30 cm above a padded surface by two identical columns. Attached at each end of the beam was a shaded goal box. Mice were placed on the beam in a perpendicular orientation to habituate, and were then monitored for a maximum of 60 sec. The number of foot slips each mouse has before falling or reaching the goal box was recorded for each of three successive trials. The average foot slips for all four trials was calculated and recorded. Errors are defined as foot slips and recorded both numerically and using Feeney scores. To prevent injury from falling, a soft foam cushion was always kept underneath the beam. Animals that fell off were placed back in their position prior to the fall.

### Rotarod

The animal was placed onto the rod (diameter 3.6 cm) apparatus to assess differences in motor coordination and balance by measuring fore- and hind limb motor coordination and balance (Rotarod 7650 accelerating model; Ugo Basile, Biological Research Apparatus, Varese, Italy). This procedure was designed to assess motor behavior without a practice confound. The animals were habituated to the apparatus by receiving training sessions of two trials, sufficient to reach a baseline level of performance. Then the mice were tested a further 3 times, with increasing speed. During habituation, the rotor rod was set at 1.0 rpm, which was gradually raised every 30 sec, and was also wiped clean with 30% ethanol solution after each session. A soft foam cushion was placed beneath the apparatus to prevent potential injury from falling. Each animal was tested for three sessions, with each session separated by 15 min, and measures were taken for latency to fall or invert (by clinging) from the top of the rotating barrel.

### Radial Arm Maze

Spatial learning was evaluated using an eight-arm radial maze with a water well at the end of each arm. Clear Plexiglas guillotine doors, operated by a remote pulley system, controlled access to the arms from a central area from which the animals entered and exited the apparatus. After 4 days of adaptation to the maze, water-restricted mice (2 h daily access to water) were given one training session per day for ten consecutive days. We use this relatively long adaptation period as we have found that these Tg AD mice tend to be very anxious and will not run the maze well without adaptation [24]; [27]. Prior to each day's testing, the mice were adapted to the room with lights on for 15 min. For each session, all arms were baited with saccharine flavored water, and animals were permitted to enter all arms until the eight rewards had been consumed. The number of errors (entries to previously visited arms) and time to complete each session were recorded.

### F) Antibody levels

Antibody levels were determined in duplicate on 1∶100 dilutions of plasma using ELISA as described previously [27]; [28], in which 50 µg/plate Aβ1-42, pABri or purified PHF was coated onto Immulon 2HB 96 well microtiter wells (Thermo, Waltham, MA). The bound antibodies were detected by a horseradish peroxidase labeled goat anti-mouse IgG (Amersham Biosciences, Piscataway, NJ) or a peroxidase conjugated goat anti-mouse IgM (Sigma; A8786). Tetramethyl benzidine (TMB; Pierce, Rockford, IL) was the color developing substrate and the readings were done at 450 nm.

### G) Histology

Mice were anesthetized with sodium pentobarbital (150 mg/kg, i.p.), perfused transaortically with phosphate buffer, and the brains processed as described previously [27]; [29]. The right hemisphere was immersion-fixed in periodate-lysine-paraformaldehyde, whereas the left hemisphere was snap-frozen for measurements of Aβ levels. Serial coronal sections (40 µm) were cut, and every fifth section (30–40 sections in total) was stained with a mixture of 4G8/6E10, monoclonal antibodies that recognizes Aβ and stains both pre-amyloid and Aβ plaques [24]; [30]. In addition two series of sections were immunostained with anti-GFAP and anti-CD11b antibodies. GFAP is a component of the glial intermediate filaments that forms part of the cytoskeleton and is found predominantly in astrocytes. CD11b is a member of the β-integrin family of adhesion molecules; also known as MAC-1 or complement receptor 3 [CR3]) and is commonly used as a marker for microglia [31]; [32]. Immunostaining was performed as described previously [24]; [30]. Briefly, sections were incubated in 6E10/4G8 each at a 1∶1000 dilution in PBS-T for 3 h. A mouse-on-mouse immunodetection kit (Vector Laboratories, Burlingame, CA) was used. The sections were incubated first with biotinylated anti-mouse IgG secondary antibody for 1 h at a 1∶2000 dilution and later with the avidin-peroxidase complex for 30 min at the same dilution. The sections were then reacted in 3,3-diaminobenzidine tetrahydrochloride with nickel ammonium sulfate (Ni; Mallinckrodt, Paris, KY) color intensification solution. Immunohistochemistry of 6E10/4G8 immunolabeled tissue sections was quantified with a Bioquant image analysis system (BIOQUANT Image Analysis Corporation, Nashville, TN), and unbiased sampling was used [30]. All procedures were performed by an individual blinded to the experimental conditions of the study. The cortical area analyzed was dorsomedial from the cingulate cortex and extended ventrolaterally to the rhinal fissure within the right hemisphere. The area of the grid was 800 µm^2^×800 µm^2^, and Aβ deposit load was measured in 20 cortical frames per mouse (640×480 µm^2^ each) chosen randomly. The threshold of the Aβ immunoreactive areas is set so that areas of <5 µm in diameter are not counted. This is done so that small artifactual areas of staining are not counted and intra-neuronal immunoreactivity is also not counted. With the latter caveat, the Aβ burden is defined as the percentage of area in the measurement field occupied by reaction product.

GFAP staining (polyclonal, 1∶1000; 3 h, Dako, Denmark) was performed with a primary antibody diluent composed of 0.3% Triton X-100, 0.1% sodium azide, 0.01% bacitracin, 1% bovine serum albumin (BSA), and 10% normal goat serum in PBS, and secondary biotinylated goat anti-rabbit antibody (Vector) reacted for 1 h at 1∶1000 dilution. CD11b immunohistochemistry (rat anti-mouse, 1∶500; 3 h, Serotec) was performed similarly to that for GFAP staining except that the secondary antibody was goat anti-rat (Vector) diluted 1∶1000. Reactive astrocytosis (GFAP immunoreactivity) was rated on a scale of 0–4. The rating was based on a semiquantitative analysis of the extent of GFAP immuoreactivity (number of GFAP immunoreactive cells and complexity of astrocytic branching), as we have previously published [27]; [30]. The assessment of the CD11b immunostained sections was based on a semiquantitative analysis of the extent of microgliosis (0, no microglia; 1, a few resting microglia; 2, a few ramified and/or phagocytic microglia; 3, moderate number of ramified/phagocytic microglia; 4, numerous ramified/phagocytic microglia), as we have previously reported [27]; [30].

Plasma from vaccinated mice with the highest titer to Aβ1-42 and PHF, as well as the pre-immune (T0) plasma from the same mice (as a control), was used for immunostaining of human tissue. Staining was performed on 8 µm deparaffinzed sections of temporal cortex. Tissue samples were of subjects from the New York University Alzheimer's Disease Center Brain Bank, who fulfilled the National Institute on Aging-Reagan criteria for AD or were age matched controls with no AD related pathology (or other neuropathology) at autopsy. Selected series were double-immunostained with pooled plasma from pABri immunized Tg mice and either PHF-1 mAb (to abnormally phosphorylated tau protein [33]) or anti-Aβ using a mixture of monoclonal 4G8 and 6E10 [30]. Briefly, sections were incubated overnight at 4°C with pooled plasma from the T6 bleedings of pABri immunized Tg mice diluted 1∶100 in PBS, 0.1% Triton X-100, 0.01% sodium azide and 1% BSA. Bound antibody staining was performed with a secondary biotinylated goat anti-mouse IgM (mu chain specific, Vector Laboratories) incubated for 1 h at a 1∶500 dilution. First, primary antibody staining was revealed with 3,3′-diamonobenzidine (DAB Sigma-Aldrich) and nickel ammonium sulfate intensification. After several washes, the tissue was blocked again in PBS 10% FBS, 0.2% Triton X-100 and the second primary antibody PHF-1 (monoclonal, 1∶200; 1 h) or a mixture of anti-Aβ 4G8/6E10 (1∶1000 each, 1 h) was added, followed by incubation with alkaline phosphatase labeled horse anti-mouse IgG (1∶500, 1 h; Vector Laboratories). Next an alkaline phosphatase substrate kit (Vector) was applied to produce a red reaction product.

In addition to using non-AD tissue as a control of specific immunoreactivity with plasma from pABri vaccinated mice, we absorbed the immune plasma with aggregated/oligomeric Aβ1-42 for immunostaining.: In order to prepare aggregated Aβ for absorption, 1 mg of synthetic Aβ 1–42 was dissolve in 200 µL of 1,1,1,3,3,3 hexafluoro-2-propanol (HFP) (Sigma). The mixture was let stand at room temperature until the solvent was evaporated, and 1 mL of 50 mM TRIS pH 7.2 was added under sterile conditions. The mixture was kept at room temperature for month until clear fibrils could be seen. Electron microscopic examination of this preparation demonstrated it to be a mixture of mainly Aβ1-42 fibrils, and some oligomers (data not shown). The mixture was thoroughly vortexed before aliquoting for absorption. For absorption, 1 mL of a 1∶100 dilution in TBS of an immune serum from successfully vaccinated animals was mixed with 200 µL of the aggregated Aβ 1-42, rotated for 1 hour at room temperature and then overnight (for at least 16 hours) at 4°C. The mixture was centrifuged at 4°C, at 14,000×g for 15 minutes and the supernatant separated and used as absorbed serum for immunohistochemical staining as described above. The pellet composed of aggregated Aβ1-42 was washed twice with 0.5 mL of TBS, centrifuged at 14,000× g for 10 minutes and the supernatants pooled and marked as washes. The pellet was then incubated twice for 5 minutes with 300 µL aliquots of 0.1 M glycine, pH 2.5, vortexed and centrifuged for 10 minutes at 14,000×g. The supernatants were pooled and immediately brought to pH 7.4 with a 1 M TRIS, pH 10 solution, and used as eluted antibodies for immunostaining as described above.

### H) Tissue homogenization and sandwich ELISA assay for soluble Aβ levels

Extraction of Aβ from brain tissue was performed as described [30]. Brains were weighed and homogenized (10% w/v) in homogenization buffer, 20 mM Tris, 250 mM sucrose, 1 mM EDTA, 1 mM EGTA with freshly prepared 100 mM phenylmethylsulfonyl fluoride, 5 µg/ml pepstatin A and a protease inhibitor cocktail (Complete, Roche Diagnostics, Indianapolis, IN). For extraction of soluble Aβ, brain homogenates were thoroughly mixed with an equal volume of 0.4% diethylamine (DEA)/100 mM NaCl, then spun at 135,000× g for 1 hour at 4°C, and subsequently neutralized with 1/10 volume of 0.5 M Tris, pH 6.8. The samples were then aliquoted, flash-frozen on dry ice, and stored at –80°C until loaded onto ELISA plates. Similarly for extraction of the total Aβ, 200 µl of each homogenate was added to 440 µl of cold formic acid (FA) and sonicated for one minute over ice. Subsequently, 400 µl of the solutions were spun at 100,000 g for 1 hour at 4°C. 210 µl of the resulting supernatants were diluted with 4 ml of FA neutralization solution (1 M Tris, 0.5 M Na_2_HPO_4_, 0.05% NaN_3_), aliquoted, flash-frozen on dry ice and stored at −80°C until used for Aβ measurements.

The total and soluble Aβ levels were measured using a combination of mouse monoclonal antibody 6E10 (specific to an epitope present on amino acid residues 1 to 16 of Aβ) and two different rabbit polyclonal antibodies specific for Aβ40 (R162) and Aβ42 (R165), in a double-antibody sandwich ELISA as described previously [24]; [30]. The optical density (OD) was measured at 450 nm. The relationship between OD and Aβ peptide concentration was determined by a four-parameter logistic log function. Non-linear curve fitting was performed with the KinetiCalc program (Biotek Instruments, Inc., Winooski, VT) to convert OD of plasma to estimated concentrations. The assay was performed by an investigator blinded to group assignment. The levels of Aβ species are presented as µg of Aβ per g of wet brain, taking into account dilution factors introduced by multiple steps throughout the assay (brain homogenization and extraction procedures).

### I) Western Blot analysis of Aβ oligomers

For Western immunoblot analysis, 10% w/v brain homogenates were centrifuged at 25,000 g for 10 min at 4°C, and the supernatants were transferred to clean tubes and stored as previously described [24]; [30]. The total protein concentration in the supernatant was determined using the Bicinchoninic acid assay (BCA; Pierce, Rockford, IL). Samples (40 µg of total protein), were mixed with an equal volume of Tricine sample buffer (BioRad, Hercules, CA), were electrophoresed on 12.5% Tris-tricine polyacrylamide gels, under nonreducing conditions and transferred to nitrocellulose membranes. The blots were blocked with 5% nonfat dry milk in TBS-T, pH 8.3, for 2 hours at room temperature, then incubated with oligomer-specific rabbit A11 polyclonal antiserum (Biosource, Camarillo, CA), diluted 1∶1000 in TBS-T, 0.1% BSA for 2 hrs at room temperature. Bound antibody was detected after 1 hr incubation with horseradish peroxidase-conjugated goat anti-rabbit IgG 1∶8000 (Pierce, Rockford, IL) and the ECL detection system (Pierce, Rockford, IL). The specificity of A11 staining was confirmed by Western blots of the same samples using anti-Aβ monoclonal antibodies 6E10 or 4G8 as previously described [24]. Densitometric analysis of A11 immunoreactive oligomer specific bands was performed with NIH Image J version 1.34 software.

### J) Statistical analysis

Data from the radial arm maze were analyzed by two-way repeated measures ANOVA followed by a Neuman-Keuls *posthoc* test. Differences between groups in the amyloid burden, Aβ levels within the brain and levels of oligomers, were analyzed using a Student's unpaired two-tailed *t-*test. Data from the GFAP and CD11b immunostaining quantitation were analyzed by one-way ANOVA followed by a Neuman-Keuls *posthoc* test. Statistical analysis was done using GraphPad Prism version 5.0 (GraphPad Software Inc., La Jolla, CA).

## Results

### A) Characterization of the aggregation state of the pABri and its conformation

**Figure 1:**
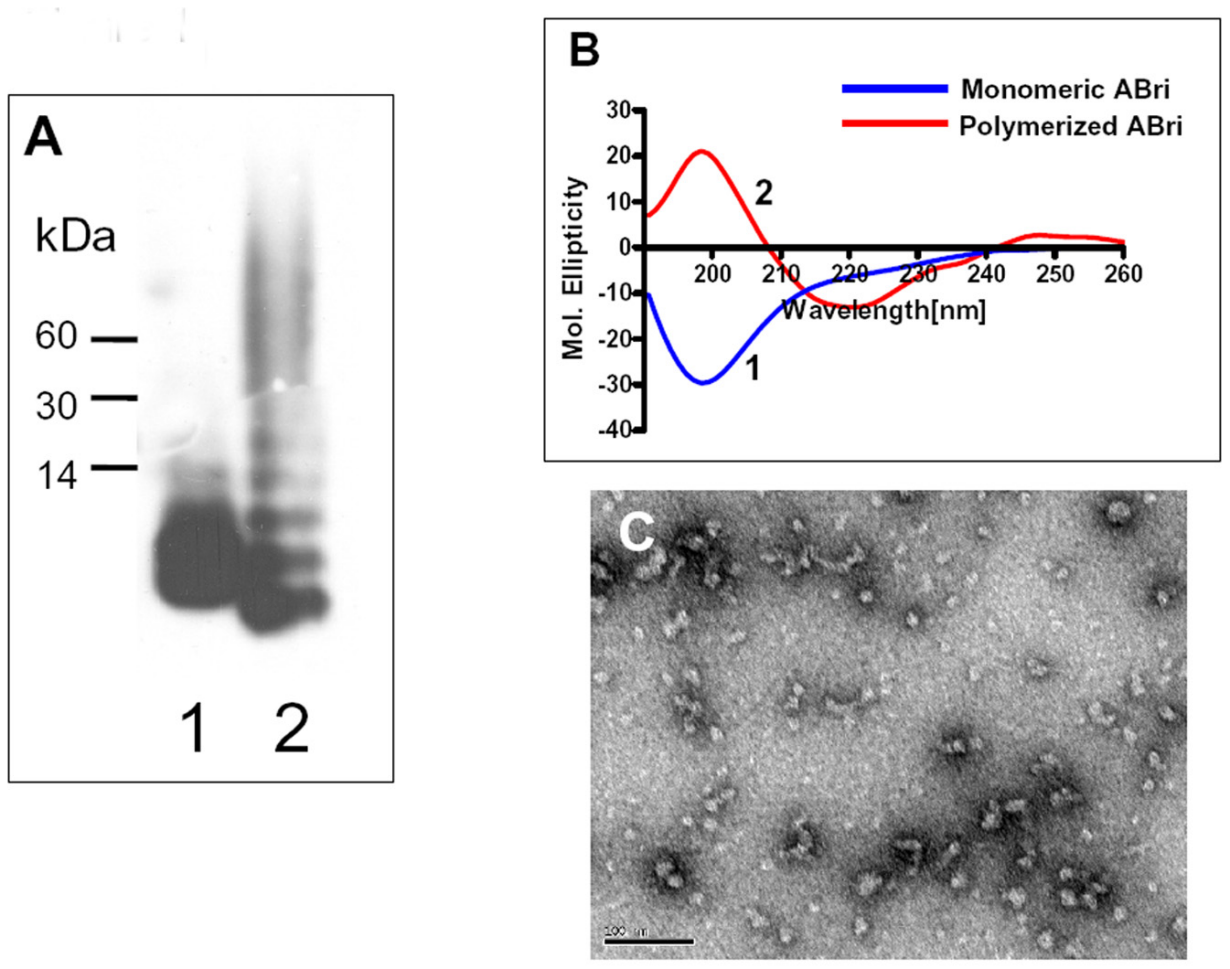
Characterization of polymerized ABri. A Western blot using specific polyclonal anti-ABri antisera is shown in panel A. The freshly dissolved ABri peptide was run in lane 1. This preparation of ABri is mainly monomeric with some lower order aggregates of dimers and tetramers in contrast to the polymerized ABri peptide which has less monomer with a predominance of higher order aggregates in a range of 30 to 100 kDa (lane 2). Shown in panel B is the circular dichroism of these peptides. The freshly dissolved ABri peptide has a predominant random coil structure with a minimum at 195 nm, in contrast to the polymerized ABri peptide that has a predominant β-sheet structure with a minimum at 220 nm and a maximum at 195 nm. In panel C an electron micrograph of negatively stained polymerized ABri peptide is shown, which is predominately in the form of spherical particles of ∼10 nm (Scale bar, 100 nm).

As determined by SDS-PAGE and Western blotting the freshly dissolved ABri peptide is mainly monomeric with some lower order aggregates of dimers and tetramers (Figure 1A, lane 1). The pABri had a lower percentage of monomeric form, an increase of multimers and a predominance of higher order aggregates in a range of 30 to 100 kDa (Figure 1A, lane 2). The apparent molecular weight of pABri monomers appears to be lower then those of the fresh sample, most likely reflecting the differing conformational states of these peptides and the amount of bound SDS. Circular dichroism of these peptides indicated that the freshly dissolved ABri peptide has a predominant random coil structure with a minimum at 195 nm, in contrast to the pABri that had a predominantly β-sheet structure with a minimum at 220 nm and a maximum at 195 nm (Figure 1B). The electron microscopic appearance of the pABri is predominately spherical particles of ∼10 nm, similar to what has been reported for other amyloid oligomers (Figure 1C) [34]; [35]. In contrast the mainly monomeric, freshly dissolved ABri peptide did not form any structures such as spherical particles or fibrils by electron microscopy (data not shown).

### B) Antibody titers

**Figure 2:**
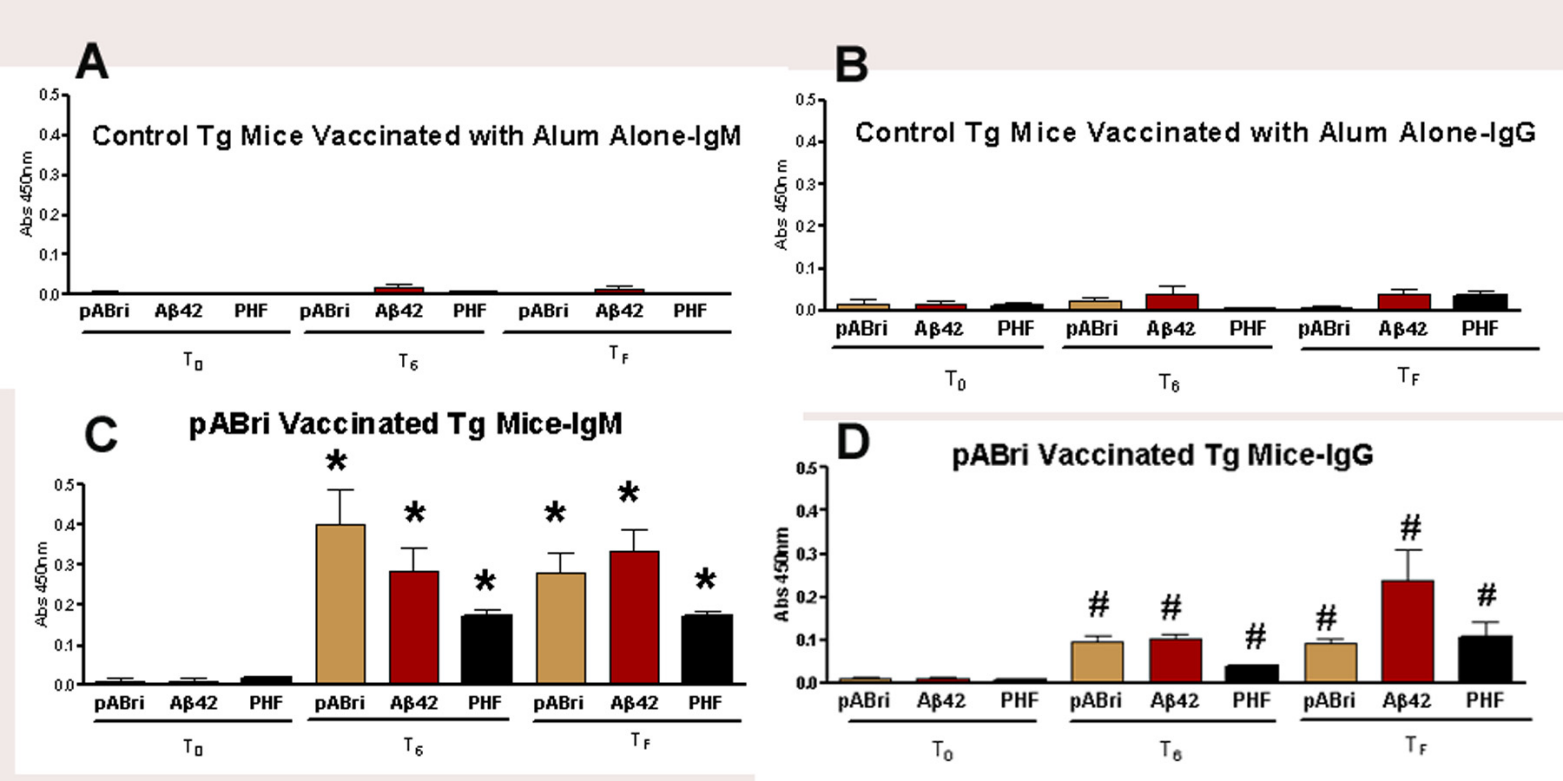
Antibody levels in control and pABri vaccinated mice. Shown are bar graphs of the IgG and IgM antibody levels against polymerized ABri, Aβ42 and PHF at T0, T6 and TF. In A and B, titers of IgM and IgG in controls are shown, respectively. In C and D, titers of IgM and IgG in pABri vaccinated mice are shown, respectively (*p<0.0001, #p<0.01).

In vehicle control mice there were no significant titers to pABri, Aβ1-42 or purified PHF (Figure 2A and B). In the pABri vaccinated mice, significant IgG and IgM titers were noted against Aβ42, polymerized ABri and purified PHF (p value by unpaired t-test was p<0.0001 and p<0.01 for IgM and IgG respectively for both the bleedings at T6 and TF) (See Figure 2C and D).

### C) Sensorimotor and Cognitive Testing

**Figure 3:**
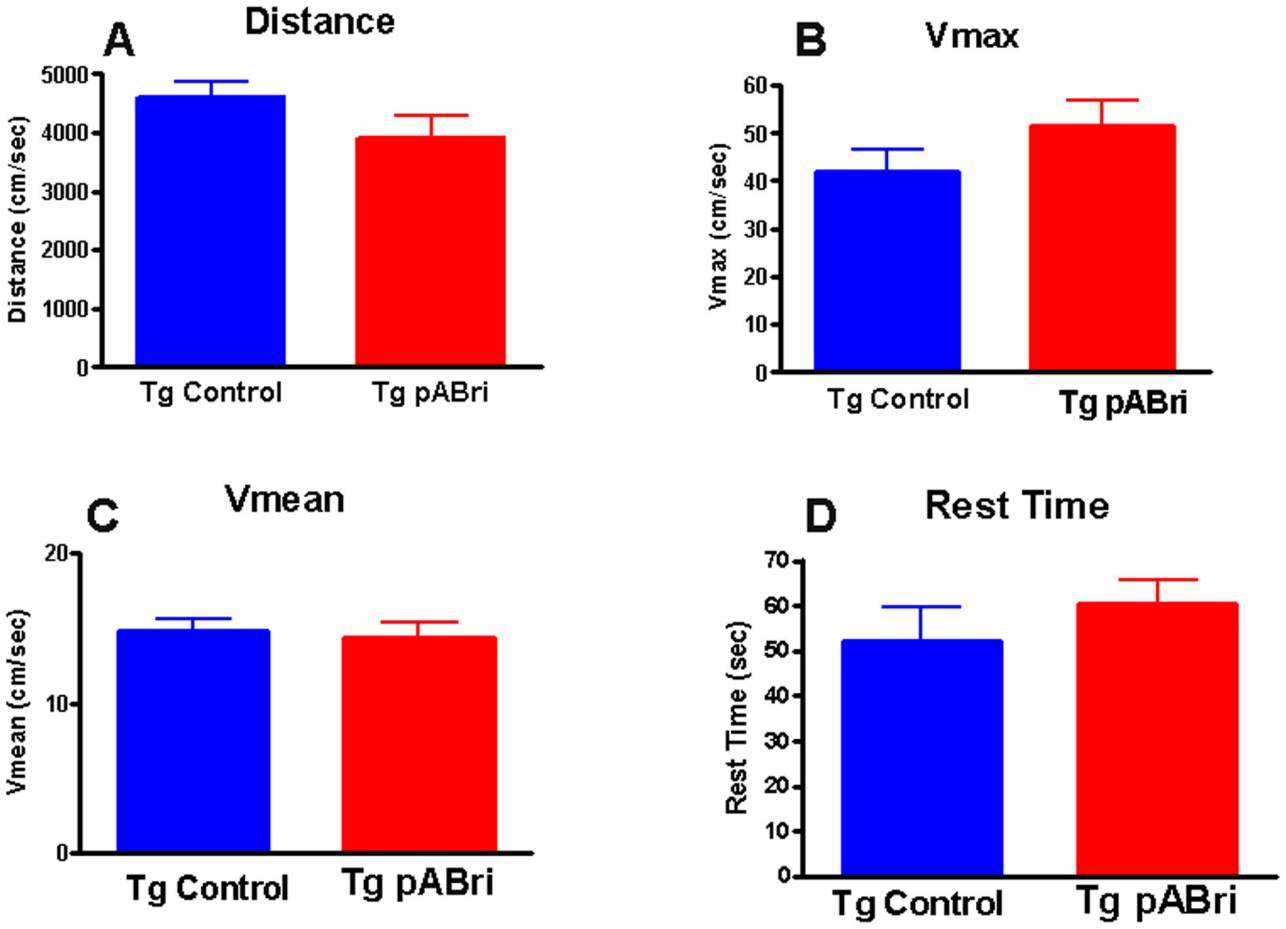
Locomotor activity testing. Shows results of locomotor activity testing comparing Tg control and Tg pABri vaccinated mice. No significant differences were noted in distance traveled, maximum velocity (Vmax), mean velocity (Vmean) or in resting time.

In order to verify that cognitive testing was not confounded by differences in sensorimotor abilities in the ABri vaccinated versus control mice, sensorimotor testing was conducted first. There were no significant difference between the groups in locomotor activity (see Figure 3), traverse beam testing and rotarod testing (data not shown).

**Figure 4:**
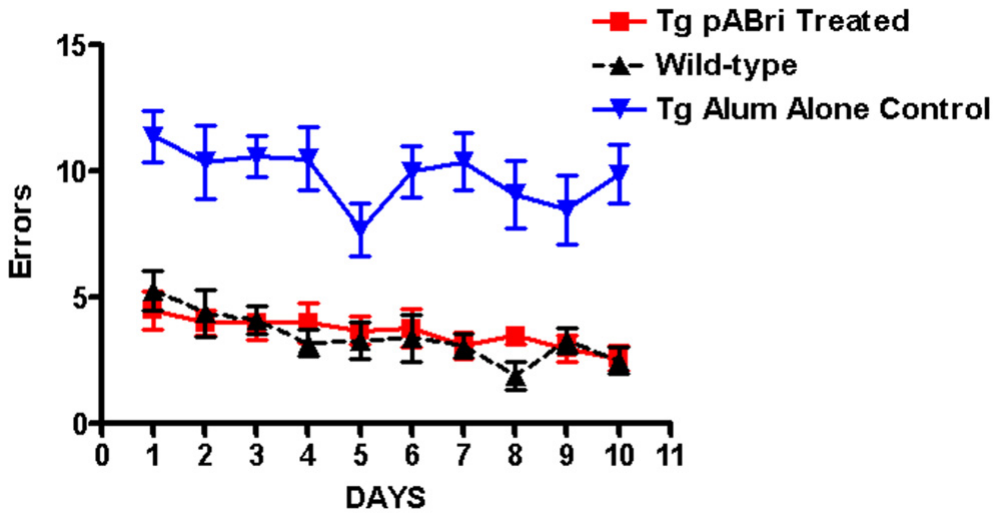
Cognitive testing on a radial arm maze. The number of errors is plotted versus the day of testing. There are statistically significant differences between the untreated control Tg mice versus the pABri treated Tg mice and wild-type controls. By two-way ANOVA the treatment effect was p<0.0001. Post-hoc Neuman-Keuls testing indicated that both the wild-type controls and pABri vaccinated Tg mice were significantly different from the control Tg mice (p<0.001). There are no differences between the wild-type controls and the pABri treated Tg mice.

Radial arm maze cognitive testing showed there were statistically significant differences between the untreated control Tg mice versus the treated Tg mice and wild-type controls (Figure 4). By two-way ANOVA the treatment effect was p<0.0001 and the day effect was p = .01. Post-hoc Neuman-Keuls testing indicated that both the wild-type controls and treated Tg mice were significant different from the control Tg mice (p<0.001). There was no difference between the wild-type controls and the treated Tg mice. The mean error rate on day 1 was 4.5 and 5.3 versus 2.6 and 2.5 on day 10 in the treated Tg mice (p<0.05, by two-tailed t-test) and the wild-type controls (p<0.01, by two-tailed t-test), respectively, indicating that both groups showed learning over time. In the Tg non-treated controls the mean day 1 error rate was 11.4 versus 9.9 on day 10 (no statistically significant difference), indicating that these mice did not learn over time.

### D) Amyloid Quantitation by Stereology and Biochemical Analysis

**Figure 5:**
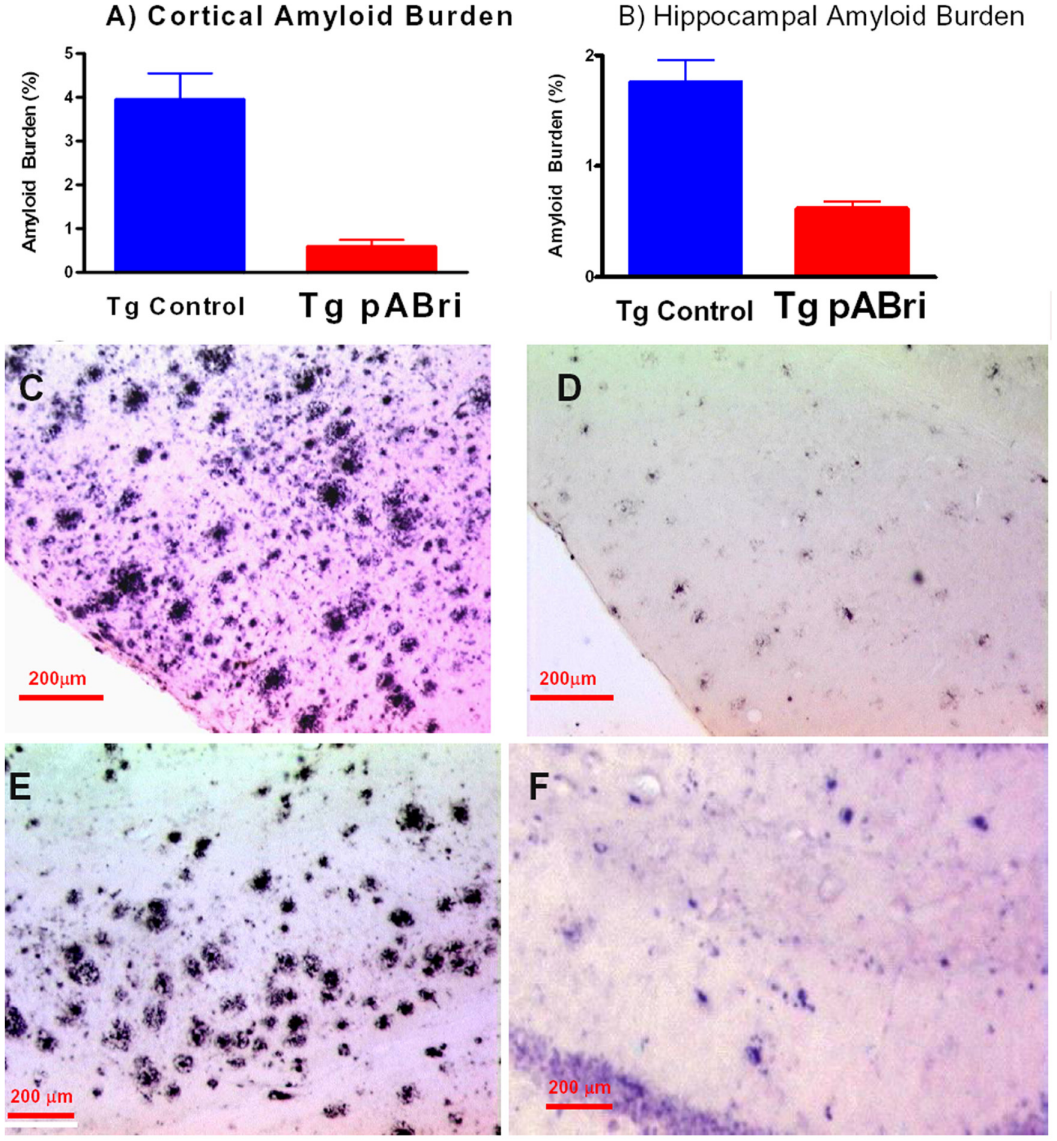
Quantitation of amyloid burden in the cortex and hippocampus. A) Shows a bar graph of the amyloid quantitation by stereology in the cortex and hippocampus of Tg control and pABri vaccinated Tg mice. There were significant reductions in the amyloid burden (% area occupied by 4G8/6E10 immunoreactivity) in both the cortex (85% reduction) and hippocampus (65% reduction); p = 0.0001 and p = 0.0002, respectively. C-F show representative immunostained sections with anti-Aβ antibodies 4G8 and 6E10 in the cortex (C and D) and in the hippocampus (E and F) of control Tg mice (C and E) and pABri vaccinated mice (D and F).

There were significant reductions in the amyloid burden (% area occupied by 4G8/6E10 immunoreactivity) in both the cortex (85% reduction) and hippocampus (65% reduction); p = 0.0001 and p = 0.0002, respectively (see Figure 5A and B). The amyloid burdens we calculated in these Tg mice are similar to what we and others have previously reported [24]; [26]; [36]–[38]; however, the burdens are less than in other reports [39]. This variability is likely related to differing experimental procedures between laboratories. Representative immunostained sections are shown in Figure 5C-F (scale bar  = 200 µm).

**Figure 6:**
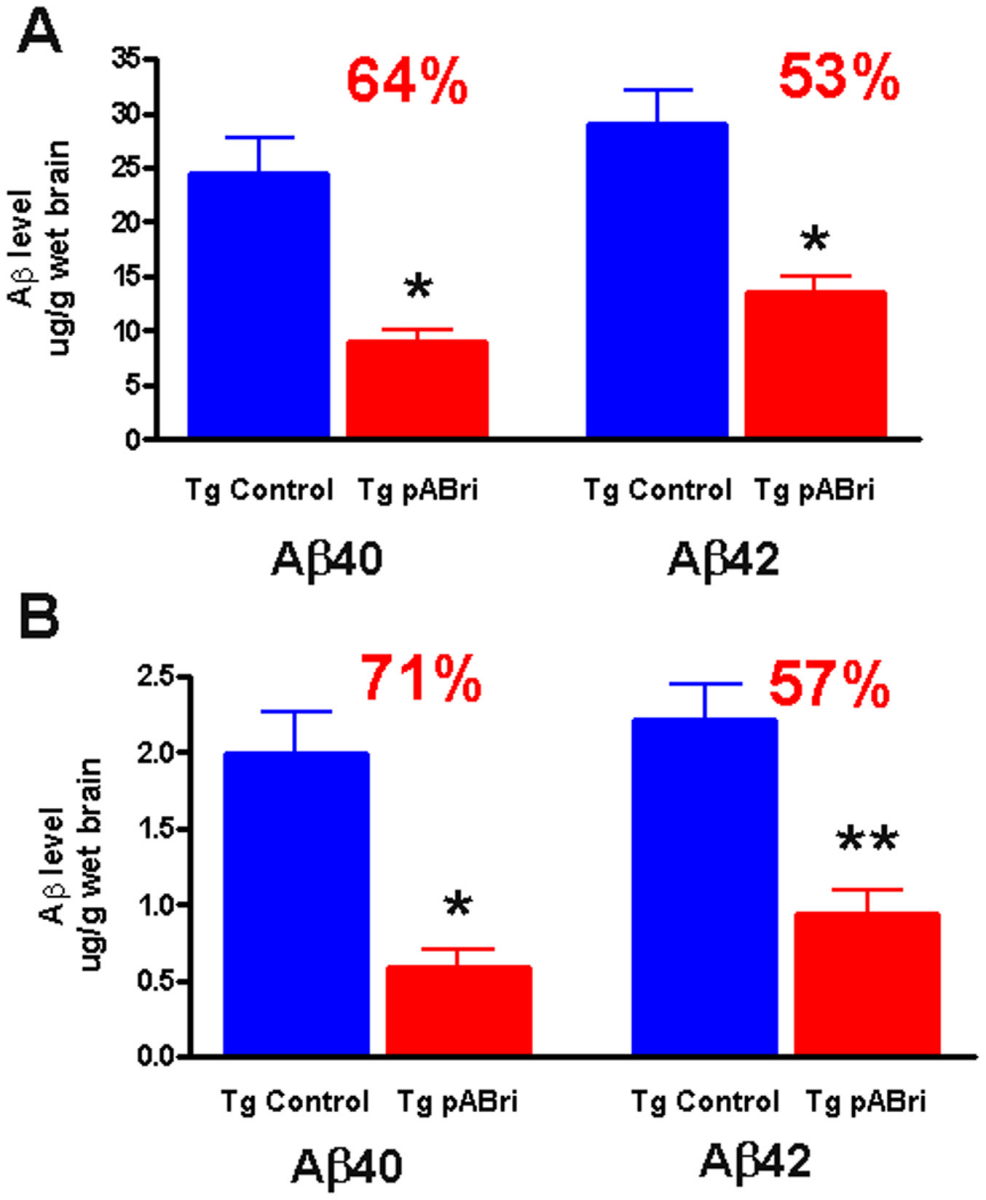
Levels of Aβ40 and Aβ42 in the formic acid and DEA extracted material from brains of control Tg and pABri vaccinated Tg mice. A) In the formic acid extract fraction Aβ40 and Aβ43 were reduced 64% and 53%, respectively (*p<0.0001) in the vaccinated mice. B) In the DEA extracted fraction Aβ40 (*p<0.0001) and Aβ42 (**p = .0002) were reduced by 71% and 57%, respectively in the vaccinated mice.

Significant reductions in the biochemically extracted Aβ40 and Aβ42 levels were also noted (Figure 6). In the formic acid extract fraction Aβ40 and Aβ42 were reduced 64% and 53%, respectively (p<0.0001). In the DEA extracted fraction Aβ40 (p<0.0001) and Aβ42 (p = .0002) were reduced by 71% and 57%, respectively, (p values are by unpaired, two-tailed t-tests).

### E) Immunostaining of AD tissue by plasma from immunized mice

**Figure 7:**
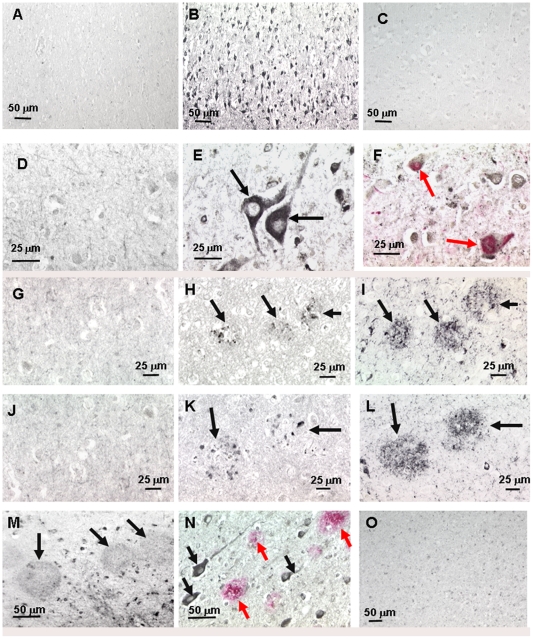
Immunostaining of AD tissue by plasma from immunized mice. Panel A shows immunolabeling using plasma (T6) from pABri vaccinated Tg mice on normal, aged brain sections. No specific immunolabeling is seen. In AD temporal cortex sections extensive cytoplasmic neuronal immunoreactivity is evident, shown in B. Absorption of the immune plasma with aggregated Aβ1-42 resulted in loss of neuronal immunolabeling, shown in C. D shows a higher magnification of the lack of specific labeling with plasma from a pABri vaccinated Tg mouse in normal brain tissue. In E a higher magnification of the neuronal cytoplasmic and dendritic immunolabeling is shown (see arrows). F depicts doubling labeling where the neuronal staining with the immune plasma is seen in black and neurofibrillary tangle immunolabeling with PHF-1 is seen in red (see arrows). Hence some neurofibrillary tangles immunolabeled with both PHF-1 and the plasma from pABri vaccinated mice. In G immunostaining with plasma which had been absorbed with aggregated Aβ1-42 on an AD temporal cortex section is shown. H shows immunolabeling in a sequential section to G using plasma which had been eluted from the aggregated Aβ1-42 pellet. I shows immunolabeling with PHF-1 in a sequential section. The arrows show labeling of dystrophic neurites with the eluted immune mouse plasma in H and with PHF-1. J, K and L depict a similar series of pictures using a different plasma sample from a Tg mouse immunized with pABri, showing a lack of specific labeling in J, immunolabeling of dystrophic neurites with the eluted plasma sample in K and the matching PHF1 labeling in L. Rare amyloid β plaques also showed faint immunolabeling with plasma from pABri immunized mice, as shown in M (see arrows). N shows a double immunolabeling where the plasma from a pABri immunized mouse is labeling neuronal perikarya and dendrites (see black arrows) and Aβ immunoreactivity is seen in red (see red arrows). There is no co-localization of the plasma immunolabeling with the anti-Aβ 4G8/6E10 immunolabeling. O depicts a lack of specific immunolabeling at a lower magnificent of a similar temporal cortex AD section to M and N where the pre-immune (T0) plasma was used from the same mouse.

Plasma (T6) from pABri vaccinated Tg mice gave no immunolabeling in normal, aged brain sections (Figure 7A). In AD temporal cortex sections, extensive cytoplasmic neuronal immunoreactivity is evident (Figure 7B). Absorption of the immune plasma with aggregated Aβ1-42 resulted in loss of neuronal immunolabeling (Figure 7C). Figure 7D shows a higher magnification of the lack of specific labeling with plasma from a pABri vaccinated Tg mouse on normal brain tissue. In Figure 7E a higher magnification of the neuronal cytoplasmic and dendritic immunolabeling is shown (see arrows). Figure 7F depicts doubling labeling where the neuronal staining with the immune plasma is seen in black and neurofibrillary tangle immunolabeling with PHF-1 is seen in red (see arrows). Hence some neurofibrillary tangles immunolabeled with both PHF-1 and the plasma from pABri vaccinated mice. In Figure 7G immunostaining with plasma which had been absorbed with aggregated Aβ1-42 on an AD temporal cortex section is shown. Figure 7H shows immunolabeling in a sequential section to G using plasma which had been eluted from the aggregated Aβ1-42 pellet. Figure 7I shows immunolabeling with PHF-1 in a sequential section. The arrows show labeling of dystrophic neurites with the eluted immune mouse plasma in Figure 7H and with PHF-1 in Figures 7I. Figures 7J, K and L depict a similar series of pictures using a different plasma sample from a Tg mouse immunized with pABri, showing a lack of specific labeling in J, immunolabeling of dystrophic neurites with the eluted plasma sample in K and the matching PHF-1 labeling in L. Rare amyloid β plaques also showed faint immunolabeling with plasma from pABri immunized mice, as shown in Figure 7M (see arrows). Figure 7N shows a double immunolabeling where the plasma from a pABri immunized mouse is labeling neuronal perikarya and dendrites (see black arrows) and Aβ immunoreactivity is seen in red (see red arrows). There is no co-localization of the plasma immunolabeling with the anti-Aβ 4G8/6E10 immunolabeling. Figure 7O depicts a lack of specific immunolabeling at a lower magnificent of a similar temporal cortex AD section to Figures 7M and N where the pre-immune (T0) plasma was used from the same mouse. This pattern of staining is similar to what has previously been reported with an anti-Aβ oligomer polyclonal mouse sera [40].

### F) Quantitation of Aβ oligomers

**Figure 8:**
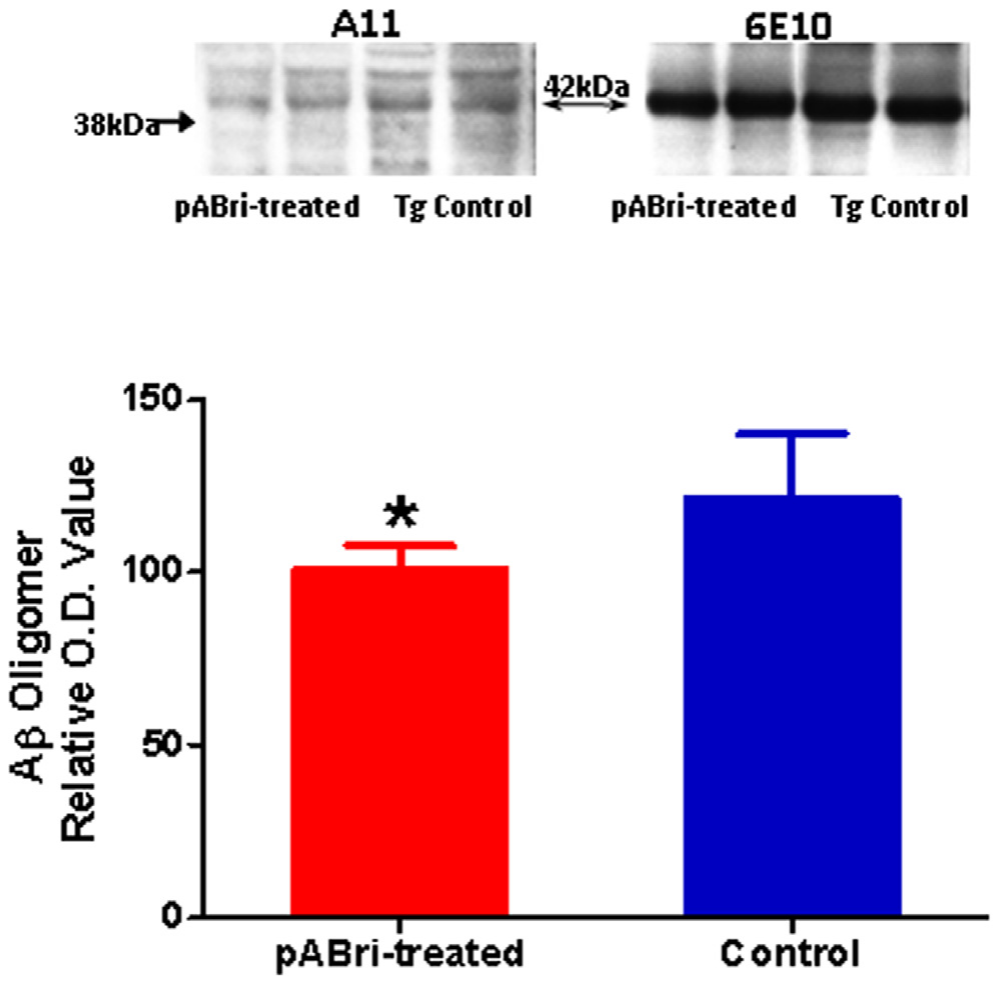
Densitometric quantitation of soluble oligomeric Aβ. The levels of pathogenic Aβ oligomers in the brain homogenates were assessed by Western-blot using the A11 oligomer-specific antibody as illustrated on top left of the figure for two representative brain extracts from two pABri vaccinated and two control Tg mice.The A11 ∼42 kDa immunoreactive band was also immunoreactive with anti-Aβ 6E10 monoclonal antibody (top right of figure). No oligomers were detected in wild-type mice (data not shown). A bar graph of the densitometric analysis of the A11 immunoreactive band is shown at the bottom of the figure (two-tailed *t-*test, *p<0.05).

Soluble oligomeric Aβ ligands (also known as ADDLs) may account for memory loss and AD neuropathology, thus presenting a significant therapeutic target [41]. The level of pathogenic Aβ oligomers in the brain homogenates were assessed by Western-blot using the A11 oligomer-specific antibody (Figure 8, top left). The A11 immunoreactive band obtained band was ∼42 kDa, similar to what we have previously reported [24]. This band was chosen for analysis as it also immunoreacted with anti-Aβ monoclonal antibody 6E10 (Figure 8, top right) and was not present in wild-type mice (data not shown). pABri vaccination led to a significant decrease in the levels of A11 immuno reactive (42 kDa) oligomers (two-tailed *t-*test, p<0.05; Figure 8, bottom).

### G) Assessment of Astrocystosis and Microglial Activation

**Figure 9:**
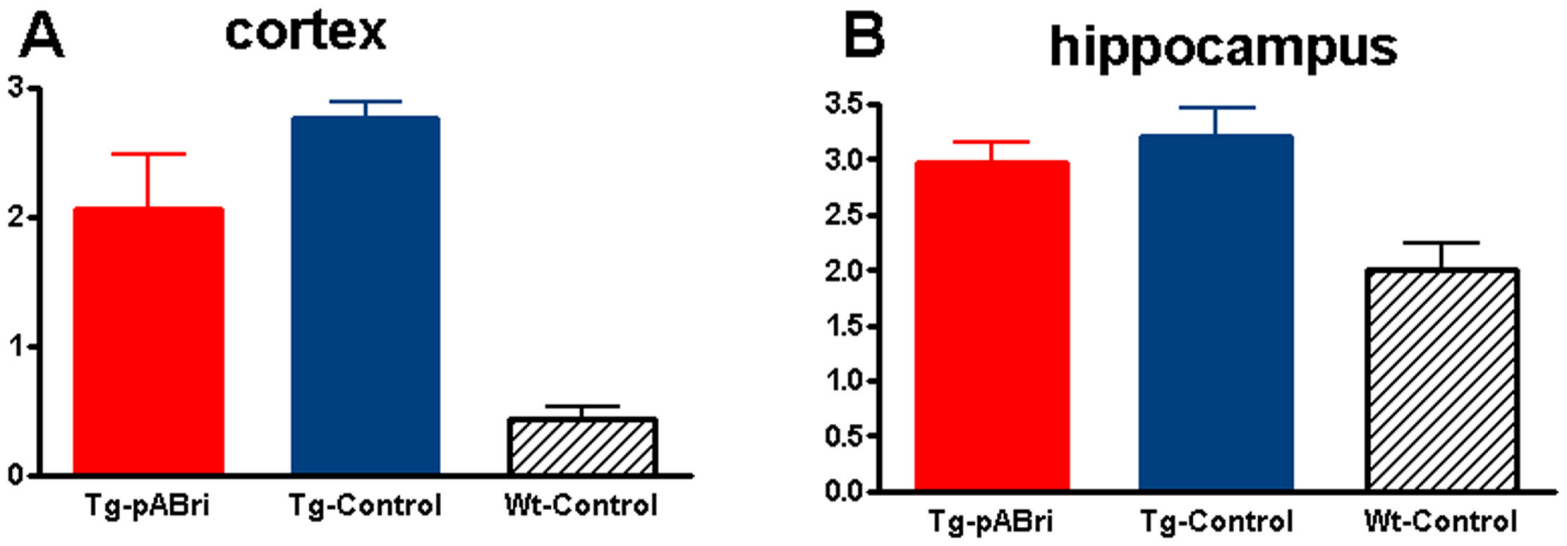
Quantitation of Astrocytosis. Reactive astrocytosis was analyzed semiquantitatively on a scale of 0–4 [30]. There was a non-statistically significant trend for a reduction in GFAP immunoreactivity in the cortex of pABri treated Tg mice versus Tg controls. Wild-type controls had significant less GFAP immunoreactivity then either Tg mouse group (wild-type controls versus either Tg pABri or Tg controls, p<0.01 and p<0.05 in the cortex and hippocampus, respectively).

GFAP immunoreactivity in the cortex and hippocampous in control Tg mice versus the treated Tg mice did not differ significantly, although there was a non-statistically significant trend for a reduction of GFAP immunoreactivity in the cortex of treated Tg mice (Figure 9A and B). As expected wild-type control mice had less GFAP immunoreactivity than either Tg mouse group (Newman-Keuls posthoc test, wild-type controls versus either Tg controls or Tg treated mice p<0.01 and p<0.05 in the cortex and hippocampus, respectively).

**Figure 10:**
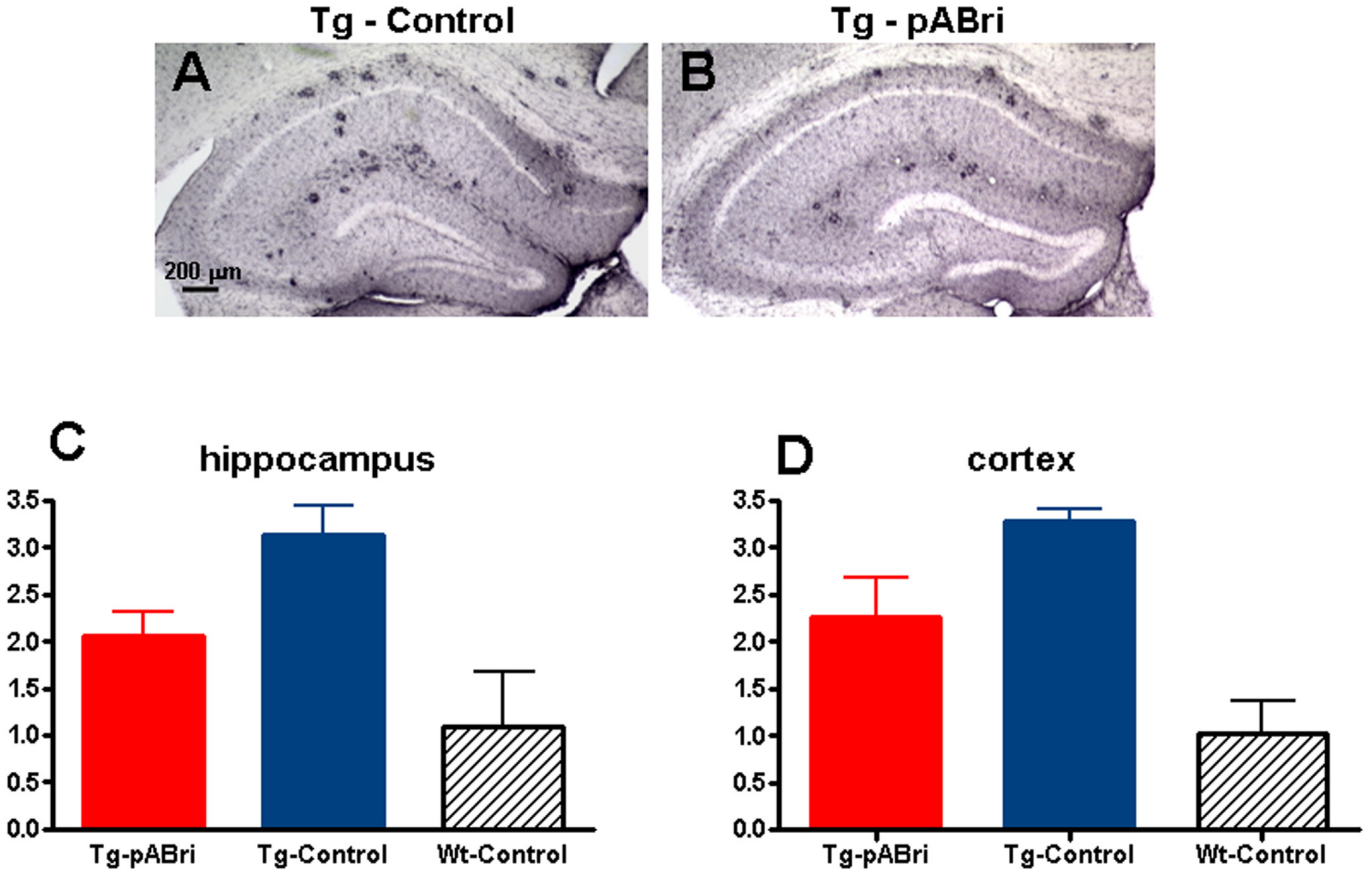
Quantitation of microgliosis by CD11b immunoreactivity. pABri treatment reduced CD11b immunoreactivity compared to Tg controls in both the hippocampus and cortex. A and B show representative sections of the hippocampus in a Tg control (A) and a pABri treated Tg mouse (B). C shows semiquantitative analysis of CD11b in the hippocampus (p<0.05 pABri Tg versus Tg controls; p<0.05 wild-type controls versus Tg controls). D shows semiquantitative analysis of CD11b in the cortex (p<0.05 pABri Tg versus Tg controls; p<0.01 wild-type controls versus Tg controls). Wild-type controls did not differ significant from pABri Tg mice in either the cortex or the hippocampus, although there was a trend for controls to have less CD11b immunostaining.

CD11b immunostaining was performed. CD11b is a well-established microglial and mononuclear phagocyte marker [31]. The assessment of microglial marker CD11b was based on semiquantitative analysis of the extent of microgliosis (Figure 10). pABri treatment resulted in an overall hippocampal and cortical reduction in CD11b immunoreactivity (Newman-Keuls posthoc test p<0.05 pABri Tg versus Tg controls in both hippocampus and cortex).

pABri treated mice did not differ significantly by posthoc testing from wild-type controls, although there was a clear trend for wild-type mice to have less CD11b immunoreactivity. As expected, wild-type mice differed significantly from Tg controls (Neuman-Keuls posthoc test p<0.05 in the hippocampus and p<0.01 in the cortex).

## Discussion

In the present study we demonstrate for the first time that immunization in an AD Tg mouse model with a foreign peptide in a polymerized, β-sheet rich form is able through conformational mimicry to induce an immune response to both Aβ42 and PHF. The immunogen we used corresponds to the 13 amino acids of the carboxyl end of the amyloid that is deposited in British amyloidosis, where a missense mutation in a stop codon results in the transcription of a novel intronic sequence [16]; [17]; [42]. This peptide has no homology to known mammalian proteins, but is highly amyloidogenic [17]; [43]. As expected, the pABri we used also induced an immune response to its primary sequence. Although antibodies to primary amino acid sequences tend to produce strong reactions with their ligands, this immune response is very unlikely to be associated with autoimmune toxicity since normal mice (and other mammals) do not express this peptide as part of any protein. In fact, this 13 amino acid sequence would only be found in the very rare individuals who are affected with British amyloidosis. This immune response is associated with a strong behavioral rescue in the treated Tg mice as they performed similar to wild type mice in the radial arm maze. A marked difference was noted on the radial arm maze from day 1 of the testing. This is likely related to 4 days of adaptation to the maze that all the mice had, during which period the wild-type and treated Tg mice were presumably able to learn the maze partially. Both the wild-type controls and the treated Tg mice showed significant learning comparing the error rate from day 1 to day 10, while the Tg non-treated controls showed no significant learning. The testing of this memory task was not confounded by any differences in the sensorimotor activity between the control and vaccinated mice as shown by the locomotor activity, rotarod and traverse beam testing. Moreover, the behavioral rescue was associated with a marked reduction in the amyloid burden as determined histologically and biochemically. The anti-Aβ42 titers our vaccinated mice developed were relatively modest; however, as we and others have suggested, behavioral rescue is most closely linked to Aβ oligomer reductions and not with either the degree of amyloid plaque deposition reduction or overall anti-Aβ titer [27]; [44]; [45]. Hence, it is not the absolute degree of the humoral response generated but its quality in terms of effective targeting of toxic species that is most important. The most toxic forms of Aβ are thought to be oligomeric [41] and importantly we showed that our approach was associated with a reduction of these toxic species of Aβ. When we used the plasma from pABri immunized mice to immunolabel control and AD brain tissue, we show that neuronal perikarya and dystrophic neurites were stained. This is similar to the pattern previously reported with another anti-oligomer mouse polyclonal plasma [40]. Presumably this represents the immunodetection of both intracellular oligomeric Aβ which has been documented in neurons both biochemically and by microscopic examination [46]; [47], as well as aggregated, abnormal tau. The specificity of this reaction was verified by absorption using aggregated Aβ1-42, as well as the lack of immunolabeling on normal brain tissue. We were also able to elute the absorbed immune plasma from the aggregated Aβ1-42 pellet and show specific labeling of dystrophic neurites. It is likely that our immunization procedure with pABri lead to an immune response to a variety of abnormal oligomer and amyloid fibril related conformational states, and it is this combination which led to the behavioral rescue along with the marked reduction in amyloid burden.

The majority of past active and passive immunization studies in mouse models and all the past trials in humans have used an approach where both the normal conformer (sAβ) and the pathological conformer (Aβ) are targeted. This is an important short coming as interfering with normal sAβ will inhibit its physiological functions such as neuroprotection, modulation of long term potentiation and innate immunity [48]–[50]. In the initial human trial of active vaccination the use of a self-antigen as an immunogen (Aβ42) was associated with encephalitis in some 6% of patients [11]. This remains an issue with on-going human trials of passive immunization where a proportion of participants have developed what has been called vasogenic edema [51]. We document in the present study that the treatment with pABri in the Tg mice led to a significant overall reduction of microglial activation as judged by CD11b immunoreactivity, with a trend for a reduction in GFAP immunoreactivity. This is consistent with our use of pABri as an immunogen which is not associated with excessive CNS inflammation. We hypothesize that an active immunomodulatory approach that, as in our studies, uses an immunogen that does not correspond to a self-antigen, but through conformational mimicry is able to induce a response that recognizes pathological conformers, will be much less likely to be associated with autoimmune toxicity.

Another significant drawback of the current immunization approaches tested in humans is that targeting only Aβ related pathology significantly reduces Aβ plaques without evidence of a corresponding significant behavioral rescue in results presented so far [51]; [52]. The limited autopsy data from the initial human active vaccination trial targeting Aβ42 showed that patients had partial or near complete plaque removal and a reduction of Aβ load compared to age matched non-immunized controls. However, there were no differences between placebo and active immunization groups in the long-term survival outcome, time to severe dementia and in cognitive outcome measurements such as ADAS-Cog, MMSE or DAD [52]. In living patients part of a passive immunization trial targeting Aβ, a 25% amyloid reduction versus controls was documented using PET imaging studies, in the absence of measurable cognitive benefits [51]. This suggests that to attain an effective immunotherapeutic approach the targets should include all forms of Aβ toxic conformers and the tau related pathology. Our conformational mimicry immunomodulatory approach demonstrated that an immune response was evident both against Aβ and PHF pathological forms, as shown by ELISA measurements and tissue staining of human AD sections. Normal human brain sections did not show any immunolabeling with the plasma from pABri vaccinated mice indicating a specificity for pathology associated protein conformers.

In summary we documented a novel active immunization approach using pABri in a β-sheet rich conformation that targets an abnormal conformation that is shared by aggregated/oligomeric Aβ and PHFs. We hypothesize that this type of immunomodulatory approach may produce interference or disruption of β-sheet structures in multiple neurodegenerative diseases associated with pathologic protein conformers.

## References

[1] Perl DP (2010). Neuropathology of Alzheimer's disease.. Mt Sinai J Med.

[2] Jellinger KA (2009). Recent advances in our understanding of neurodegeneration.. J Neural Transm.

[3] Rostagno A, Holton JL, Lashley T, Revesz T, Ghiso J (2010). Cerebral amyloidosis: amyloid subunits, mutants and phenotypes.. Cell Mol Life Sci.

[4] Schneider JA, Arvanitakis Z, Bang W, Bennett DA (2007). Mixed brain pathologies account for most dementia cases in community-dwelling older persons.. Neurol.

[5] van der ZJ, Sleegers K, Van BC (2008). Invited article: the Alzheimer disease-frontotemporal lobar degeneration spectrum.. Neurol.

[6] Josephs KA, Whitwell JL, Knopman DS, Hu WT, Stroh DA (2008). Abnormal TDP-43 immunoreactivity in AD modifies clinicopathologic and radiologic phenotype.. Neurol.

[7] Wisniewski T, Golabek AA, Kida E, Wisniewski KE, Frangione B (1995). Conformational mimicry in Alzheimer's disease. Role of apolipoproteins in amyloidogenesis.. Am J Pathol.

[8] Giasson BI, Forman MS, Higuchi M, Golbe LI, Graves CL (2003). Initiation and synergistic fibrillization of tau and alpha-synuclein.. Science.

[9] Wisniewski T, Boutajangout A (2010). Immunotherapeutic approaches for Alzheimer's disease in transgenic mouse models.. Brain Struct Funct.

[10] Wisniewski T, Chabalgoity JA, Goni F (2007). Is vaccination against transmissible spongiform encephalopathies feasible?. OIE Sci Tech Rev.

[11] Gilman S, Koller M, Black RS, Jenkins L, Griffith SG (2005). Clinical effects of Aβ immunization (AN1792) in patients with AD in an interupted trial.. Neurol.

[12] Lambert MP, Velasco PT, Viola KL, Klein WL (2009). Targeting generation of antibodies specific to conformational epitopes of amyloid beta-derived neurotoxins.. CNS Neurol Disord Drug Targets.

[13] Lee EB, Leng LZ, Zhang B, Kwong L, Trojanowski JQ (2006). Targeting amyloid-beta peptide (Abeta) oligomers by passive immunization with a conformation-selective monoclonal antibody improves learning and memory in Abeta precursor protein (APP) transgenic mice.. J Biol Chem.

[14] Moretto N, Bolchi A, Rivetti C, Imbimbo BP, Villetti G (2007). Conformation-sensitive antibodies against alzheimer amyloid-beta by immunization with a thioredoxin-constrained B-cell epitope peptide.. J Biol Chem.

[15] Mamikonyan G, Necula M, Mkrtichyan M, Ghochikyan A, Petrushina I (2007). Anti-A beta 1-11 antibody binds to different beta-amyloid species, inhibits fibril formation, and disaggregates preformed fibrils but not the most toxic oligomers.. J Biol Chem.

[16] Vidal R, Frangione B, Rostagno A, Mead S, Revesz T (1999). A stop-codon mutation in the BRI gene associated with familial British dementia.. Nature.

[17] Rostagno A, Tomidokoro Y, Lashley T, Ng D, Plant G (2005). Chromosome 13 dementias.. Cell Mol Life Sci.

[18] Holcomb L, Gordon MN, McGowan E, Yu X, Benkovic S (1998). Accelerated Alzheimer-type phenotype in transgenic mice carrying both mutant amyloid precursor protein and presenilin 1 transgenes.. Nature Med.

[19] Vidal R, Barbeito AG, Miravalle L, Ghetti B (2009). Cerebral Amyloid Angiopathy and Parenchymal Amyloid Deposition in Transgenic Mice Expressing the Danish Mutant Form of Human BRI(2).. Brain Pathol.

[20] Sadowski M, Pankiewicz J, Scholtzova H, Ripellino JA, Li Y (2004). Blocking the apolipoprotein E/ß-amyloid interaction reduces β-amyloid toxicity and decreases β-amyloid load in transgenic mice.. Am J Pathol.

[21] Toumadje A, Alcorn SW, Johnson C (1992). Extending CD spectra of proteins to 168 nm improves the analysis for secondary structures.. Anal Biochem.

[22] Sreerma N, Woody RW (1993). A self-consistent method for the analysis of protein secondary structure from circular dichroism.. Anal Biochem.

[23] Kascsak RJ, Tonna-DeMasi M, Fersko R, Rubenstein R, Carp RI (1993). The role of antibodies to PrP in the diagnosis of transmissible spongiform encephalopathies.. Dev Biol Stand.

[24] Sadowski M, Pankiewicz J, Scholtzova H, Mehta P, Prelli F (2006). Blocking the apolipoproteinE/Amyloid β interaction reduces the parenchymal and vascular amyloid-β deposition and prevents memory deficit in AD transgenic mice.. Proc Natl Acad Sci (USA).

[25] McGowan E, Sanders S, Iwatsubo T, Takeuchi A, Saido T (1999). Amyloid phenotype characterization of transgenic mice overexpressing both mutant amyloid precursor protein and mutant presenilin 1 transgenes.. Neurobiol Dis.

[26] Scholtzova H, Wadghiri YZ, Douadi M, Sigurdsson EM, Li Y (2008). A NMDA receptor antagonist leads to behavioral improvement and amyloid reduction in Alzheimer's disease model transgenic mice shown by micro-magnetic resonance imaging.. J Neurosci Res.

[27] Asuni A, Boutajangout A, Scholtzova H, Knudsen E, Li Y (2006). Aβ derivative vaccination in alum adjuvant prevents amyloid deposition and does not cause brain microhemorrhages in Alzheimer's model mice.. Eur J Neurosci.

[28] Goni F, Knudsen EL, Schreiber F, Scholtzova H, Pankiewicz J (2005). Mucosal vaccination delays or prevents prion infection via an oral route.. Neurosci.

[29] Sigurdsson EM, Knudsen EL, Asuni A, Sage D, Goni F (2004). An attenuated immune response is sufficient to enhance cognition in an Alzheimer's disease mouse model immunized with amyloid-β derivatives.. J Neurosci.

[30] Scholtzova H, Kascsak RJ, Bates KA, Boutajangout A, Kerr DJ (2009). Induction of Toll-like receptor 9 signaling as a method for ameliorating Alzheimer's disease related pathology.. J Neurosci.

[31] Hickman SE, Allison EK, El Khoury J (2008). Microglial dysfunction and defective beta-amyloid clearance pathways in aging Alzheimer's disease mice.. J Neurosci.

[32] Morgan D, Gordon MN, Tan J, Wilcock D, Rojiani AM (2005). Dynamic complexity of the microglial activation response in transgenic models of amyloid deposition: implications for Alzheimer therapeutics.. J Neuropathol Exp Neurol.

[33] Otvos L, Feiner L, Lang E, Szendrei GI, Goedert M (1994). Monoclonal antibody PHF-1 recognizes tau protein phosphorylated at serine residues 396 and 404.. J Neurosci Res.

[34] Kayed R, Head E, Thompson JL, McIntire TM, Milton SC (2003). Common structure of soluble amyloid oligomers implies common mechanism of pathogenesis.. Science.

[35] Glabe CG (2008). Structural classification of toxic amyloid oligomers.. J Biol Chem.

[36] Dhenain M, Delatour B, Walczak C, Volk A (2006). Passive staining: a novel ex vivo MRI protocol to detect amyloid deposits in mouse models of Alzheimer's disease.. Magn Reson Med.

[37] Sanchez-Ramos J, Song S, Sava V, Catlow B, Lin X (2009). Granulocyte colony stimulating factor decreases brain amyloid burden and reverses cognitive impairment in Alzheimer's mice.. Neuroscience.

[38] Wisniewski T, Sigurdsson EM (2010). Murine models of Alzheimer's disease and their use in developing immunotherapies.. Biochim Biophys Acta Mol Basis Dis.

[39] Gordon MN, Holcomb LA, Jantzen PT, DiCarlo G, Wilcock D (2002). Time course of the development of Alzheimer-like pathology in the doubly transgenic PS1+APP mouse.. Exp Neurol.

[40] Ito K, Ishibashi K, Tomiyama T, Umeda T, Yamamoto K (2009). Oligomeric amyloid beta-protein as a therapeutic target in Alzheimer's disease: its significance based on its distinct localization and the occurrence of a familial variant form.. Curr Alzheimer Res.

[41] Lublin AL, Gandy S (2010). Amyloid-beta oligomers: possible roles as key neurotoxins in Alzheimer's Disease.. Mt Sinai J Med.

[42] Ghiso J, Vidal R, Rostagno A, Miravalle L, Holton JL (2000). Amyloidogenesis in familial British dementia is associated with a genetic defect on chromosome 13.. Molecular Basis of Dementia.

[43] Srinivasan R, Jones EM, Liu K, Ghiso J, Marchant RE (2003). pH-dependent amyloid and protofibril formation by the ABri peptide of familial British dementia.. J Mol Biol.

[44] Janus C, Pearson J, McLaurin J, Mathews PM, Jiang Y (2000). Aβ peptide immunization reduces behavioural impairment and plaques in a model of Alzheimer's disease.. Nature.

[45] Morgan D, Diamond DM, Gottschall PE, Ugen KE, Dickey C (2000). Aβ peptide vaccination prevents memory loss in an animal model of Alzheimer's disease.. Nature.

[46] Walsh DM, Tseng BP, Rydel RE, Podlisny MB, Selkoe DJ (2000). The oligomerization of amyloid beta-protein begins intracellularly in cells derived from human brain.. Biochem.

[47] LaFerla FM, Green KN, Oddo S (2007). Intracellular amyloid-beta in Alzheimer's disease.. Nat Rev Neurosci.

[48] Puzzo D, Privitera L, Leznik E, Fa M, Staniszewski A (2008). Picomolar amyloid-beta positively modulates synaptic plasticity and memory in hippocampus.. J Neurosci.

[49] Giuffrida ML, Caraci F, Pignataro B, Cataldo S, De BP (2009). Beta-amyloid monomers are neuroprotective.. J Neurosci.

[50] Soscia SJ, Kirby JE, Washicosky KJ, Tucker SM, Ingelsson M (2010). The Alzheimer's Disease-Associated Amyloid beta-Protein Is an Antimicrobial Peptide.. PLoS ONE.

[51] Rinne JO, Brooks DJ, Rossor MN, Fox NC, Bullock R (2010). (11)C-PiB PET assessment of change in fibrillar amyloid-beta load in patients with Alzheimer's disease treated with bapineuzumab: a phase 2, double-blind, placebo-controlled, ascending-dose study.. Lancet Neurol.

[52] Holmes C, Boche D, Wilkinson D, Yadegarfar G, Hopkins V (2008). Long term effects of Aβ42 immunization in Alzheimer's disease: immune response, plaque removal and clinical function.. Lancet.

